# Pedagogical strategies for the development of improvisation and composition in North Indian classical music

**DOI:** 10.3389/fpsyg.2025.1460158

**Published:** 2025-06-05

**Authors:** Emily Sayers

**Affiliations:** Faculty of Arts, Humanities and Education, Canterbury Christ Church University, Canterbury, Kent, United Kingdom

**Keywords:** pedagogy, improvisation, composition, North Indian classical music, ethnomusicology, cultural transmission, music education, performance analysis

## Abstract

North Indian Classical Music (NICM) provides a structured context to examine how improvisational and memory-based skills are developed through oral transmission. While improvisation is central to NICM performance, there is limited research on the pedagogical strategies that support its acquisition, particularly in relation to cognitive processes such as memory, pattern recognition, and schema development. This study analysed audio-visual recordings and fieldnotes from music lessons in both music schools and *guru-śiṣya paramparā* settings in India. Lessons were coded thematically with attention to instructional techniques, learner responses, and cognitive strategies including repetition, segmentation, and variation. Students were rarely asked to improvise spontaneously. Instead, learning focused on imitation and memorisation of modelled material, with inexact replication often leading to creative recomposition. Teachers used structured sequences of *palṭās* and *tāns* to support phrase construction, pitch accuracy, and intensification strategies. These techniques scaffolded both domain-specific (musical) and domain-general (cognitive) skills. Findings suggest that improvisational competence in NICM is developed through memorisation, structured variation, and implicit learning. The study contributes to understanding how oral traditions support cognitive development in music and highlights the need for further interdisciplinary research on learning and memory in non-notated musical systems.

## 1 Introduction

Cognitive aspects of musical learning have long been of interest to researchers in developmental psychology because of the way in which musical learning and performance use so many areas of the brain in their execution (Kraus and Chandrasekaran, 2010; Habibi et al., 2018). The cognitive processes at work when western musicians perform in a variety of different situations have been explored at length in the music education literature, however a paucity of research on learning, practice, memorisation and performance beyond the Western Classical tradition has been identified (Hallam et al., 2016). Within the field of ethnomusicology there is a growing body of work surrounding the cognitive processes of musicians from oral traditions (Pearce and Rohrmeier, 2012) which highlight the differences in cognitive challenge between performing improvised music when contrasted with memorised music or reading music from written notations. This study seeks to build on and develop these ideas relating to North Indian Classical Music (NICM).

Similarly, in the field of music education, the challenges that western musicians face in developing improvisation skills after they have learnt music using pedagogies common to the western classical style of learning are well documented (Kratus, 1991; Sudnow, 2002; Sawyer, 2007; Shevock, 2018). Hadar and Rabinowitch (2023) propose that factors affecting the ‘tightness’ or ‘looseness’ of improvisation within a musical style can be understood in terms of their varying levels of structural sparseness, flexible social roles, cultural nonconformity and creative freedom resulting in a continuum of overall looseness with free jazz at one end of the spectrum and western classical music at the other. This demonstrates how important contextual aspects are when attempting to understand improvisation within a style of music. The article continues to argue that NICM occupies an area of the spectrum closer to western classical music than free jazz and is an area for western classical musicians to consider when seeking to broaden their pedagogical strategies for the development of improvisation skills.

This study therefore aims to identify what the improvisatory objects and processes being used in the training and performance of NICM are. This will be achieved by reviewing ethnomusicological literature and analysing video recordings of lessons (*tālīm*). For readers unfamiliar with the context of NICM, the next few sections of the introduction provide a review of what ethnomusicologists have written about improvisation in NICM so far, with a focus on *dhrupad*, *ṭhumrī* and *ḵẖayāl* (vocal music styles). Appendix A provides a glossary of key terms at the end of this article for reference.

## 2 Materials and methods

The lack of comprehensive contemporary literature reviews on improvisation in North Indian classical music (NICM) necessitates an explanation of the improvisatory objects and processes at work in a performance. Once these have been identified, a short-term, naturalistic, qualitative research design will be employed to identify how these are taught in practice. The theoretical perspective underpinning this research design is interpretivist and it is situated within a social constructivist view of epistemology. The methods of data collection were mainly observations recorded as fieldnotes and videos. Analysis of the data has incorporated aspects of thematic analysis (Braun and Clarke, 2022) leading to schema analysis following the initial coding stages. By looking at these strategies in depth, the aim is to uncover ways that teachers develop the capabilities of their students in relation to the creation of musical performances.

This supports the overall aim to understand the multi-layered complexities of the teaching and learning strategies used using qualitative rather than quantitative methods (Cohen et al., 2018, p. 434). The study seeks to avoid simplifications through the selection of a qualitative methodology to reflect the “contradictions, richness, complexity, connectedness, conjunctions and disjunctions” (Cohen et al., 2018, p. 288) of the social, musical and educational worlds of the research participants.

Researcher positionality requires me to locate my views, values and beliefs in relation to the research process and the research output. To allow me to do this effectively, it was necessary for me to become a learner of NICM so that I could reflect on the activities that I was observing with the insight of a learner (albeit an adult, Western learner). My positionality as a researcher working in an inter-disciplinary way makes it important to ensure that in designing an appropriate methodology and set of methods by which to investigate pedagogical strategies for improvisation in NICM, that the customary approaches of both ethnomusicology and educational psychology were followed. I attempted to carefully manage the issue of me being a white, western educated, middle aged, female teacher-researcher taking my values and biases to modern India. The issues here affect not only the validity and reliability of my research but the ethics of my doing the research in the first place. Three issues in educational research in postcolonial context are highlighted by McKeever:

“Do I, as a white person have any right to research Indian experience? […] The ethics of knowledge production in a country that has experienced colonization and the dangers of conducting research that perpetuates a colonialist ethics.” (McKeever, 2000, pp. 102–103)

There were undoubtedly aspects of my privilege that were unavoidable, but I attempted to mitigate this by presenting myself authentically—as an interested student with a passion for learning from the teachers and students within the music schools, *gharānās* and *gurukuls* where I was permitted access. I felt that I had a responsibility to try not to make students or teachers feel uncomfortable due to my presence, and I attempted to convey this through my dress, my manner and responding to any conventions of behaviour that were expected of students.

Data was generated by observing lessons in six urban centres in India (New Delhi, Varanasi, Kolkata, Bhopal, Lucknow, and Mumbai) during the academic year 2016–17 as part of my PhD research. Initially, records of these were kept using written fieldnotes, but this technique progressed to making dual-perspective video recordings at a later stage in the research once codes and themes were becoming clear in the data. This allowed me to focus one camera on student(s)/*śiṣya(s)* and one camera on teachers/*gurus* which were later combined within the same frame. The written-up fieldnotes and video observations were imported into NVivo—a Computer Aided Qualitative Data Analysis Software (CAQDAS) package which allowed me to assign codes to the data, apply classifications and run queries related to the data generated (Lewins and Silver, 2007).

Participants and field sites were selected by ease of access. Whilst this is not an ideal system in terms of obtaining the widest possible spread of teachers/*gurus*, it did ensure that the collection of data was efficient, and that teachers and *gurus* were willing to give their consent to be recorded. More selective methods posed challenges, due to the presence of gatekeepers who, without a formal introduction from someone they know and respect would not be willing for me to observe their teaching and talk to their pupils. In many situations I felt that teachers were giving a ‘demonstration lesson’ rather than teaching ‘normally’, and these observations have been excluded from the data corpus. Duran et al. (2011) also experienced these difficulties and resolved them in the same way. I also excluded lessons where errors in my communication had led to me observing instrumental rather than vocal lessons, because I considered that instrumental techniques for improvisation would be different from those used in vocal lessons.

Once the data from ‘demonstration lessons’ and instrumental lessons was excluded, I had 19 h and 4 min of recorded lessons/*tālīm*. Once themes had been identified and applied to the timestamps on the videos, the CAQDAS package also gave the option of moving between episodes in the video data where themes had been identified, to check and compare the places where a particular feature had been coded for. This brought structure to a previously unstructured set of data, allowed me to keep track of (and review) transcripts of lessons and allowed me to see how far I had progressed with the coding of the dataset, and to make a note of emerging notes on the data as I coded. From this data, two illustrative examples were selected, one from a music school and one from the *guru-śiṣya paramparā* (GSP—a lineage of teaching and learning where knowledge is imparted through a relationship between *guru* and *śiṣya*) from which the illustrative examples for this research article are drawn.

## 3 The practice of North Indian classical music—its grammar and oral transmission techniques

In performance, NICM shares several conventions with western classical music. Performers tune their instruments on stage at the beginning of a concert and sometimes adjust their tuning during the performance too. They perform for a quiet and seated audience, with or without amplification, depending on the venue’s size. Those seated near the front often have higher social or artistic status and may call out in admiration at particular features of the improvised performance, to which performers may respond (Clayton, 2007; Clayton and Leante, 2015). Performances typically include an accompanying drone played by an instrument such as the *tānpūrā* and/or an electronic *śruti* box, which serves as a constant reference point, allowing musicians to focus precisely on intonation during performance, rehearsal, *tālīm*, and *riyāz* (practice). A significant emphasis is placed on the accuracy of intonation, which is remarkable given the absence of fixed pitches for tones beyond the natural fourth, fifth, and octave. The other pitches can vary microtonally between *rāgs* (named melodic frameworks for improvisation) and musicians (Bor, 1999). This complexity demands that performers internalise the notes of the *rāg* they are to improvise, resulting in the practice of doing rigorous and repetitive exercises focused on intonation during the initial stages of training. These repetitive exercises (known as *palṭās*) have been extensively described in the literature (Deshpande, 1989; Magriel, 1999), but they can affect the progress and motivation of young students too, particularly when students are reliant on their own auditory perception skills to determine whether they are rehearsing them with accuracy. *Rāg* is an important concept in NICM which is difficult to define, but usually refers to a combination of the melodic mode and vocal/instrumental colour conveyed by the performer. The melodic mode is known as the *thāt* and there are many different *rāgs* in each *thāt* for example *kalyāṇ thāt* which broadly corresponds to the Lydian mode and counts *yaman*, *hindol* and *deś* amongst its *rāgs*.

Rather than performing named compositions, each part of a concert programme will focus on one *rāg*. This intense focus on pitch accuracy highlights another crucial distinction between *rāgs* and scales. A *rāg* is not merely a scale or mode; different *rāgs* may use the same pitches but in varied configurations. The characteristics distinguishing one *rāg* from another include scale, ascending and descending lines, the number of notes, emphasised notes and register, intonation, obligatory embellishments, and the intended time of performance (Jairazbhoy, 1971; Sorrell and Narayan, 1980). The inventory of melodic ingredients of a *rāg* involves the arrangement of *swars* (notes) in ascending and descending orders of pitch, tetrachord configurations, and a hierarchy of melodic importance among the notes. This includes contours and paths for moving through this configuration, the function and aesthetic effect of each *swar*, and microtones and microtonal inflections (McNeil, 2017).

Musicians ascribe different moods and feelings to specific *rāgs*, often linked to the lyrical themes of *bandiśes* (fixed melodic compositions) performed in these *rāgs*. During improvised sections, performers must evoke the emotion of the *rāg* rather than focus on individual pitches and their transitions. Performances are constructed in real-time rather than from notation, requiring extensive memorisation of both fixed elements of the composition and key phrases of the *rāg*. These key phrases, often including distinctive ornamentation, are essential for audience recognition of the *rāg*. The oral tradition in NICM is organised around these antecedent sets of phrases, and the process of weaving them together with consideration of expansion, increase, extemporisation, and gesture is crucial for producing a compelling improvised performance (Neuman, 2012). This use of set patterns aids memory and facilitates elaboration, although the terminology used by ethnomusicologists to describe this elaboration is inconsistent, and further exploration is needed to understand the cognitive processes at work during performance. For example, Neuman (2012, p. 444) uses the terms *pakaḍ, calan*, *vistār, baṛhat* (increase), *upaj* (extemporisation or variation) and *andāz* (gesture/conjecture) but these terms are not universally understood by musicians and ethnomusicologists. McNeil (2017) considers that this elaboration works at three levels *vadi bheda, chalan bheda and uccharan bheda* and conceives of fixed melodic material as ‘seed ideas’ from which improvised materials grow.

### 3.1 The classicisation of an oral tradition

Colonial efforts to modernise and categorise aspects of Indian culture significantly influenced educational practices in institutions. Reformers like Bhātkande and Paluskar sought to place music education at the heart of cultural education, especially its theoretical aspects (Bakhle, 2005). Bhātkande envisioned a democratised system of music education that would make music instruction common and universal in India (Bhātkhande, 1934). These reforms have left a lasting impact, with the creation of music colleges which continue to follow their ideas. These reformers, who were often English-educated, high-caste, middle-class Hindus, prioritised a national system of music education with a greater focus on explicit theory teaching compared to the traditional *guru-śiṣya paramparā* (a lineage of teaching and learning where knowledge is imparted through a relationship between *guru* and *śiṣya*). Modern music colleges offer qualifications like the *Sāṅgīt Vishārad*, (equivalent to a Bachelor of Music), which include extensive factual knowledge about NICM and require students to learn numerous *rāgs* for performance (Pradhan, 2009).

This structured, institutional approach focused on amassing a breadth of knowledge quickly, contrasts with the GSP, where learners might spend months on exercises in a single *rāg* under close supervision (Deshpande, 1989). Pradhan questions the ability of students at modern music colleges to retain information long-term due to the high number of *rāgs* included in institutional syllabi (Pradhan, 2009). Teacher training also faces issues, with teaching roles often given as philanthropic gestures rather than appointments being made based on pedagogical expertise. Neuman (1980) also noted that institutional teaching methods differ significantly from the GSP. However, many private music schools today adopt its principles to promote a particular educational ethos. These schools prefer to recruit their own alumni as teachers, ensuring consistency in expectations, syllabus knowledge, and pedagogical strategies. This practice reflects a modern interpretation of *paramparā* (tradition) where the collective teachings of an institution replace the traditional *guru*. Music schools often advertise the idea of a *gurukul* (training institution run by a *guru*) or GSP as integral to their teaching model. This approach has been highlighted by Krishna as “an ideology rather than a pedagogical reality” (Krishna, 2020, p. 28). In music education, the GSP emphasises enculturation over transmissive strategies, fostering a sense of music as divine rather than a marketable commodity. This approach helps preserve the nature of the tradition and sustainability, ensuring it is passed on to future generations, however the extent to which this ethos is shared by private music schools varies between institutions.

### 3.2 The improvisatory characteristics of NICM

During a performance, musicians rely heavily on memory to improvise. Nooshin (2003) theorises that memorising models during training teaches compositional principles useful for improvisation and variation. Pressing’s cognitive model of improvisation development emphasises the improved efficiency, fluency, flexibility, error correction, and expressiveness that develops with improvisational competence. This model accounts for inventiveness and coherence through specific cognitive changes:

“Increased memory store: Expanding memory of musical, acoustic, and motor aspects.

Improved memory accessibility: Building redundant relationships and aggregating constituents into larger cognitive assemblies.

Refined perceptual attunement: Enhancing sensitivity to subtle and contextually relevant information” (Pressing, 1988, p. 166).

The relationship between memory and perceptual information selection described here, which develops increasingly efficient neural pathways, is key to developing the ability to create compelling and coherent performances of NICM.

**Figure 1:**
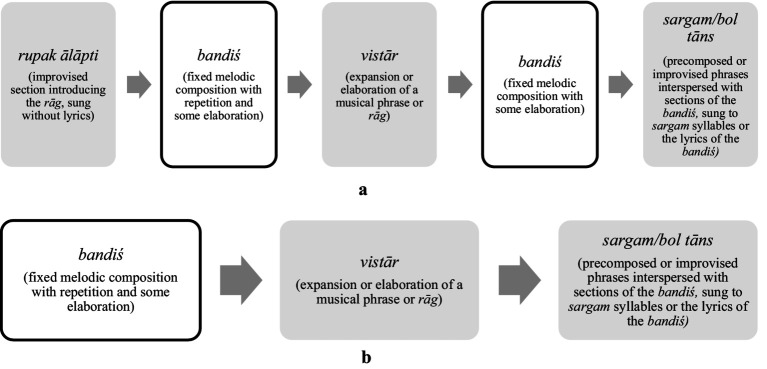
**(a)** The structure of a *baṛā ḵẖayāl* (long *ḵẖayāl*) in slow-medium tempo demonstrating the improvised sections in shaded boxes and fixed composition sections in bordered boxes. **(b)** The structure of a *choṭā ḵẖayāl* (short *ḵẖayāl*) which will often follow a *baṛā ḵẖayāl* in performance. The improvised sections are shown in shaded boxes and the fixed composition sections are shown in bordered boxes.

Despite the fact that all phrases are open for embellishment in NICM, a clear distinction is made between the composed and improvised sections of a performance (Nooshin and Widdess, 2006). The precomposed parts (*bandiśes*), vary melodically and textually between *gharānās* and have evolved over time, although their themes, lyrics, and *rāgs* have endured for centuries. Performers still ornament the *bandiś*, varying the ornamentation each time a phrase is repeated. The structure of a performance has important implications for developing improvisatory competence in NICM where a performance of a *rāg* can last for 45 min to well over an hour depending on contextual factors. The structure of a *ḵẖayāl* performance (a style of vocal music whose literal translation is imagination) is shown in Figures 1a,b. These illustrate the pre-composed and improvised sections of a *baṛā* (long) and *choṭā* (short) *ḵẖayāl*.

Whilst the length of time spent on each of these sections varies greatly, is clear from these structures that a performer will be improvising for most of the performance. Whether in terms of embellishment of precomposed material or in the creation and recreation of phrases best suited to expressing the sentiment of the *rāg*. These structures for performance featuring a balance of fixed composition and improvisation form the basis of transmission in NICM. Just as there are a balance of memorised phrases and the rearrangement, combination and creation of new musical ideas in a performance, so this mirrors the pedagogical strategies used to develop improvisatory competence. To the extent that just as a performance will begin with a focus on the drone and slow unfurling of the *ālāp* (opening section of a *baṛā ḵẖayāl*) before the more metrically bounded sections of the *bandiś*, *vistār* and *tāns*, the sections of a singing lesson are structured in just the same way.

### 3.3 Pedagogical strategies used to develop improvisatory competence

Learning *bandiśes* orally by rote puts the focus on the memory of the sounds rather than the memory of written lyrics or the degrees of the scale. This learning is approached very differently in the GSP where repertoire will be learned aurally which stands in contrast to music school teaching where written lyrics and notation will often be used for support. There are key differences between learning a song aurally and from notation. Aural learning develops strong ‘associative chains’ (Farrell, 2012) where each passage cues the memory of what comes next. This is important not just for the *bandiś* section of a performance, but also for the improvised sections that are text/rhythm oriented. In these sections, a short section of the *bandiś* (usually the first phrase, known as the *mukhṛā*) will be sung, followed by *tāns*. In some styles of music, this can present a problem, because to ‘reach for’ a specific link in the associative chain a student must start at the beginning. This is not generally an issue in NICM because the first line is returned to many times during the performance and could effectively cue a whole range of different phrases that have been modelled by a teacher and learned by rote. In contrast to this, learning from written notation/lyrics develops a student’s ‘content addressable’ memory (Chaffin et al., 2016) allowing them to answer questions such as ‘how does the second line of the *bandiś* go?’ without having to sing it in their head from the beginning. These two different ways of accessing memory demonstrate that if a student learns a *bandiś* using written notation and lyrics, these memories are more likely to be content-addressable and explicit/conscious. Learning a *bandiś* aurally will construct associative chains that are implicit/unconscious and involve procedural knowledge that cannot be easily expressed in words. This highlights the difference between learning a *bandiś* and memorising a *bandiś*, a distinction which appears in Western music but not necessarily in NICM, a context in which, to learn a *bandiś* a student must also memorise the *bandiś*.

### 3.4 Generative and formulaic perspectives on improvisation

The question of whether improvisation in NICM is generative or formulaic is contested within the literature. The generative view suggests that improvisation is driven by associative, moment-to-moment decisions based on preceding material Powers (1980) and Pressing (1988) liken this to language analysis, proposing that improvisation involves stringing together event clusters, akin to constructing a generative grammar for *rāgs*. Clarke (1988, 1993) supports this by linking improvisation to hierarchical and selective elaboration, evident in the structured progression of phrases during *ālāp*. He suggests that generative principles underpin both melody and expression, enabling performers to create infinite variations within a finite framework. In contrast to this, Powers and Widdess (2001) highlight that *rāg* improvisation differs from western tonal systems due to its integration of scale, melodic motifs, and characteristic features (*pakaḍ, calan*). This complicates generative interpretations, as improvisation often elaborates motifs through techniques like prefixing, suffixing and rhythmic manipulation rather than by assembling notes generatively. Generative elements also appear in *vistār*, where musicians construct improvisations by expanding pitch ranges and manipulating rhythmic cycles. Slawek (1998) identifies learned ‘programs’ driving this process, exemplified in both *tāns* and *vistār*. Performers balance pre-existing schemas with spontaneous adjustments, using freed cognitive resources to focus on expressive nuances.

The formulaic argument posits that improvisation relies on a repertoire of stock phrases and variable strategies adapted to the *rāg* and performance context. Zadeh (2012) critiques generative models derived from western traditions, emphasising the role of ‘stock expressions’ and ‘variable melodic outlines’ in the genre of *ṭhumrī*. These formulas, drawn from oral traditions and adapted idiomatically, serve as structural and expressive tools, guiding performers through improvisatory constraints. Ethnomusicological studies of improvisation in NICM such as Zadeh’s have contributed evidence to the formulaic argument by documenting techniques such as transposition, chromatic slides and manipulating the audience’s expectations by delaying resolutions. Magriel (1997) finds that while NICM artists from a particular *gharānā* share a common ‘dialect’, their individual ‘idiolects’ shape distinctive improvisatory styles. This explains why students often inherit stock expressions from their teachers, reflecting the transmission of formulaic knowledge.

#### 3.4.1 Improvisatory objects: ornaments/embellishments

McIntosh (1993) explores the improvisatory objects and processes involved in embellishing a *bandiś*, suggesting that improvisatory processes occur through the accumulation of components of various sizes. This process operates at different levels, including the construction of embellishments on a single note. In lessons, teachers may add ornaments, alter the rhythm of the *sthāyī*, and emphasise specific words through repetition, articulation, or gesture.

Pearson (2016) analyses both musical and physical gestures in performance, examining the relationship between *svars* (notes) and *gamaks* (ornaments). She demonstrates how musicians link these musical units coherently, likening it to the way joined-up handwriting uses different linkage patterns depending on the subsequent letter. Widdess (2014) similarly views a melodic pitch as part of a larger event, often linked into an expressive whole by glissando and other ornamentation. Pearson (2016) also introduces the term ‘coarticulation’ to describe this approach, borrowing from phonetics to explain how a phonological segment’s vocalisation is context-dependent and influenced by neighbouring sounds.

#### 3.4.2 Improvisatory objects: *palṭās*/*tāns*

Just like the *ālāp* of a performance, the sections of a performance containing *tāns* are also initially memorised. Students begin with simple *palṭā* exercises in *bilāval ṭhāṭ* (Ionian mode) which develop their memory for regular patterns of notes. They then progress to advanced *paḷās* with more unpredictable patterns, often in different *ṭhāṭs*. After mastering these, students copy *tāns* composed by their teacher. Through practice and familiarity with characteristic phrases of the *rāg*, students develop strategies to fill in gaps if they cannot remember a teacher’s phrase precisely. This is combined with composing, notating, and memorising their own *tāns*, leading to the ability to improvise their own. An interview conducted by Nicolas Magriel with *guru* Devashish Dey and his son Shubhankar Dey demonstrates these strategies and processes, highlighting how they evolve from memorising and practising difficult patterns to focusing on variation. During the interview Nicolas enquires whether the ability to sing difficult patterns relies on continued *palṭā* repetition:

**N:** but what about, not just actual tāns but do you ever do this palṭā practice,

**D:** palṭā practice, yes

**N:** just some?

**D:** it depends because in the beginning the student should practice the palṭās more but after that you must deliberately avoid them too, because doing too much of palṭā in higher stage is not only boring but the lustre of the singing is lost something.

**N:** oh, that’s interesting

**D:** so, my teacher, Kalvinji guruji, always insisted not to do alaṅkār for long a time and always to make sa sa re ga, sa re re ga, sa re ga ga, re re ga ma, re ga ga ma, re ga ma ma, ga ga ma pa… all of these things [demonstrates this by singing very fast] all these things for the younger students, once upon a time he did a lot of practice like this, two hours or one and a half hours of this kind of thing.

Once you have got that [demonstrates some more of these palṭās with gamak] already you have then to think about how to make it more beautiful rather than to make it powerful. (Dey, 2009).

The memorisation of a *palṭā* itself makes it a compositional object, but when reaching for a particular phrase, the *palṭā* training kicks in and allows a musician to grasp it. It is important to note that *palṭās* are content addressable rather than dependent on associative chains because fragments of *palṭās* need to be reassembled automatically without conscious thought. Cognitive evidence for the fact that formulaic patterns are processed more quickly and efficiently to random ones is provided by Pawley and Syder (1983).

#### 3.4.3 Improvisatory objects: formulas: stock expressions, variable melodic outlines and musical gestures

Studies of performance in NICM, particularly vocal styles, have explored the connections between spoken language skills and the ability to improvise musical phrases. As outlined in section 3.4, Zadeh (2012) critiques this application to NICM, arguing that formulas from oral literature, everyday speech, and other musical traditions serve as the building blocks of *ṭhumrī*. She highlights the difference between orality and literacy in performance, noting that formulas provide structural schemes and metrical constraints, much like in oral poetry, enabling poets to draw from a repertoire of conventional phrases rather than creating material from scratch during a performance (Zadeh, 2012, p. 38). In her thesis, Alaghband-Zadeh (2013) provides examples from Rasoolan Bai’s *ṭhumrī* performances to illustrate her point and identifies stock expressions that consistently appear in Girija Devi’s performances too. Zadeh also notes techniques for manipulating audience expectations by extending or interrupting phrases.

Artists deploy these techniques distinctively, which relates to Magriel’s (1997) ideas about dialect and idiolect, suggesting that while artists conform to dialect rules, they construct their *upaj* (improvisations on a theme) according to their personal idiolect. Once a phrase is memorised, it becomes easier to create variations and recombine different phrases imaginatively. Alaghband-Zadeh (2013) suggests that the prevalence of these formulas in performance is likely a result of how students were taught. Whether students inherit formulas implicitly or through direct instruction is crucial in analysing learning situations. Training students to listen attentively to phrases they must reproduce supports memorisation and adaptability, enabling them to ‘fill in the gaps’ where necessary. The formulas Zadeh identifies, labelled as “stock expressions,” “variable melodic outlines,” and “musical gestures,” (Alaghband-Zadeh, 2013, p. 67), along with the grand structural schemas of *ḵẖayāl*, dhrupad, and *ṭhumrī* performances, constitute improvisatory objects, as do the modal nuclei of *ālāp* and the pitch ranges of *bandiśes* (see section 3.4.5 and section 3.4.6).

#### 3.4.4 Improvisatory objects: cadential features and overall structural conventions

Cadential features such as *tihāi* (a polyrhythmic technique where a phrase is repeated three times, often used to conclude a section or performance) are also considered to be an important improvisatory object to be learnt. The strategies used to teach these are not discussed in the literature, beyond examples of students learning *tihāis* by rote and teachers composing them for their students to learn (Clayton, 2000).

Having explained that the structure of a performance contains a balance of fixed composition and improvisation, the literature also highlights how these musical structures are reflected in the structure of each lesson (*tālīm*). Where a *guru* will usually focus on the skills required for each section of the performance sequentially. For performers, having a mental map of the whole performance is crucial if one is to be able to sustain a compelling presentation of the *rāg* over 45 min or more (Snyder, 2016). The process of memorisation is expedited by the fact that whatever comes next is heavily constrained by what precedes it. Music is made easier to learn by the number of constraints placed on it, and these combine to make memory reconstruction easier. The capacity to memorise quickly improves over time (Ginsborg and Sloboda, 2007), and therefore children who have been listening to and learning these songs since childhood will find them easier to learn aurally as they get older (Magriel, 1997; McNeil, 2017; Faber and McIntosh, 2020). Sanyal and Widdess (2004) calls this concept ‘recreative ability’:

“Accustomed from birth to the sounds of his father and other family members singing and playing in the home, the young musician acquires the instinctive ability to recreate those sounds with the aid of whatever materials he has learned formally. This process of tālīm encourages the development of this recreative ability, which can also be acquired by non-family disciples, and underlies the phenomenon of ‘improvisation’ in Indian music. Recreative ability is a fundamental link between the realms of tradition and performance in Indian music: it is the mastery of processes of improvisation as well as the memorisation of fixed repertory.” (Sanyal and Widdess, 2004, p. 130)

**Figure 2:**
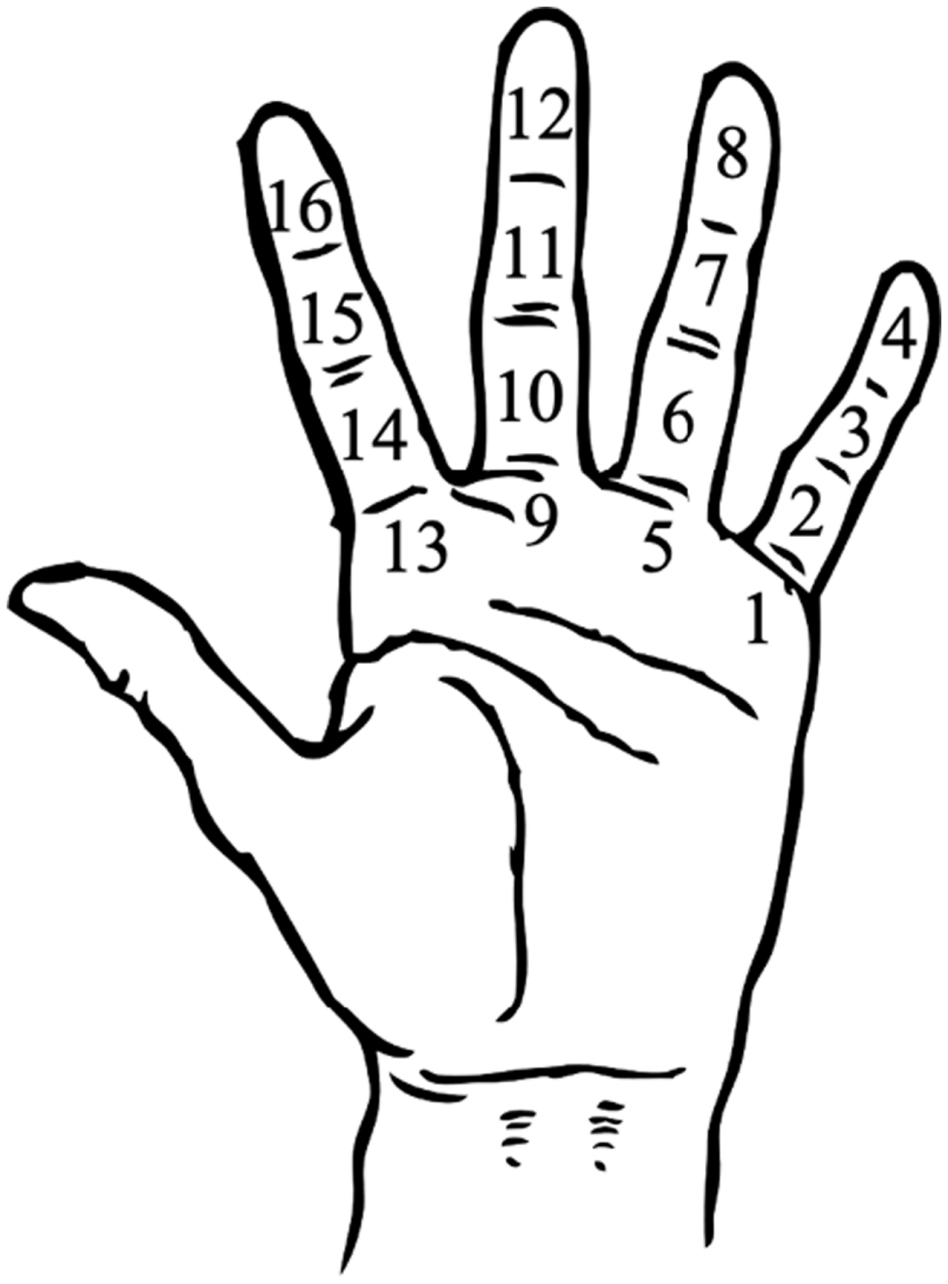
Diagram illustrating the areas of the left hand where each beat is signified with a tap from the left thumb.

The ability to embellish phrases idiomatically therefore improves over time and with repeated exposure to the performances of *gurus*. The aspect of a composition that will not vary, however, is the metrical position of the beginning of the *mukhṛā*. If the *bandiś* begins on the 13th beat of a 16 beat (*tīn tāl*) cycle, the performer will always ensure that it begins at that point, even if the variation on the previous repetition of the *mukhṛā* lasted a few beats longer. This is because, during a performance, a great deal of importance is attached to the arrival of the *sam* (first beat of the cycle), and musicians attach a high degree of importance to ensuring that improvised phrases ‘come in *tāl*’ (finish on the *sam*). This makes keeping track of the metric cycle within a performance a very important feature of children’s learning and explains why teachers ensure that students learn how to count the metric cycle on the joints of their left hand, using their left thumb to keep their place (see Figure 2).

During lessons, this strategy will be used in a variety of ways, sometimes modelled by the teacher with the addition of saying the numbers out loud, but quite often it is used spontaneously and silently by students during *tāns* to ensure that the phrase they are constructing will conclude at the start of the next metric cycle. Teachers also ensure that students learn the cheironomy of metric cycles. This is the system by which each *vibhāg* (duration of rhythmic phrasing within a *tāl*) is marked by a clap or a wave. Students will use this technique during lessons, and in performance. During lessons, it can be helpful for a student who is improvising to see these points in time marked out by their classmates. It is also common to see members of an audience in a concert setting replicating these gestures. By creating structures of this type for improvisation, students are provided with a fixed point in time when they can return to the material from the start of the associative chain, freeing up their working memory to think about the next improvisatory process or object to deploy.

The implication of *tālīm* being structured in this way means that it becomes virtually indistinguishable from performance. Zadeh describes how the structure of improvisation was taught by her teacher:

“When I was learning to sing Indian classical music, this overall pattern would inform not only the pieces my teacher taught me, but even the way she structured my lessons. We would start with long, slow exercises focusing on sa, then explore lower register, and then start a series of exercises which reached ever higher notes while increasing in speed and complexity. By learning in this way, this overall structural progression came to seem perfectly natural to me.” (Alaghband-Zadeh, 2013, p. 34)

This makes the learning process within NICM highly efficient, as there are essentially no ‘easy pieces’ that must be mastered before a student is allowed to embark on professional repertoire. This is not to say that a student will be producing professional performances from the beginning of their training, and some *rāgs* are considered more difficult to learn than others, or not suitable for very young students due to the challenges of conveying the *bhāva* (emotion) connected with the *rāg*.

#### 3.4.5 Improvisatory objects: modal nuclei

**Figure 3:**
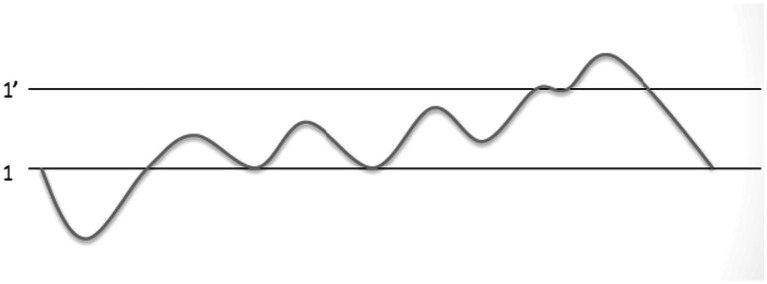
Example of the pitch range used in melodic expansion of the *ālāp* from a lecture given by Widdess (n.d.).

Modal nuclei are improvisatory objects which contribute to the constraints within which performers improvise. In the sequential phrases of the *ālāp* (the first section of a *ḵẖayāl* performance) pitches tend to lie within a specific range, developing according to a particular pattern. This strategy of using modal nuclei gradually reveals the *rāg*, ensuring that all features unique to the *rāg* are highlighted in the *ālāp* (Powers, 2001). Structuring the *ālāp* in terms of these pitch ranges also creates a teachable model for students. The *ālāp* progresses from the lowest to the highest register of a singer’s voice, with different scale degrees revealed sequentially. This progression is illustrated in Figure 3 in a diagram created by Widdess (2017), where the ‘*sa*’ of the middle octave and the ‘*sa’* of the higher octave are represented by 1 and 1′.

Rather than indicating a single melody line, each section of the line in this diagram can be divided into phases. Within each phase, a singer produces several idiomatic phrases within the specific pitch range before moving to the next phase. The diagram shows that the ‘*sa*’ of the middle register is frequently returned to until the phrases approach the upper ‘*sa*’, and at the end of the *ālāp*, there is a return to ‘*sa*’ in the middle octave. This map of pitch contours provides students and performers with yet more constraints within which to improvise.

#### 3.4.6 Improvisatory objects: pitch conventions of *bandiśes*

The idea that with practice, material becomes easier to learn because it conforms to expectations of other music that has been heard before is an important feature of learning in NICM because it also cultivates melodic expectations for *rāgs*. The idea of learning via exposure is not unique to NICM, and has been explored as one of the possible universal processes involved in learning music beyond the Western tradition. Stevens and Byron note that within a particular culture, perception becomes “attuned to or constrained by the culture-specific regularities and conventions (statistical structures, probabilities) of that environment” (Stevens and Byron, 2016, p. 26). *Śiṣyas* must develop melodic expectations for the *bandiś* to expedite aural learning of melodically complex material. Very early in training a student will become aware that the melody of the *sthāyī* (first section of a *bandiś*) moves in both the lower tetrachord of the middle octave and the lower octave. They will also develop an expectation that the *antarā* (second section of the *bandiś*) will rise to the upper tetrachord of the middle octave and the upper octave (Clayton, 2000, p. 114). Widdess refers to this as the *antarā* formula (Widdess, 1981, p. 161) and finds few exceptions to it in the published compositions and recorded performances examined.

To support students’ logical understanding of the shape of a melody, oral notation is frequently used to scaffold aspects of learning, including the *bandiś*. This oral notation is one of the transmissive strategies identified as part of the classicisation of the oral tradition in section 3.1, where teachers sing the note names of a *bandiś* in *sargam* (where pitches are referred to as *sa, re, ga, ma, pa, dha,* and *ni*) to help a student reproduce them accurately. This strategy can be linked to the concept of notational audiation (inner hearing of notated music). Woody (2012) found that this strategy helped musicians to perform on their instrument, with or without a score.

Morris (2005, pp. 52–55) considers that an important factor in developing students’ improvisatory capabilities is the number of *bandiśes* that a student knows in a particular *rāg*. He estimates that, whilst a student might know 3–6 *bandiśes* in each *rāg*, an established performer will know something like 10–30. This means that the task of learning compositions over the course of a learning and performing career, will lead to performers developing a repertoire of between 400 and 1,500 *bandiśes*. The more familiar a student is with the process of learning a *bandiś*, the faster they will be learnt, and experienced students have been known to pick up the lyrics and melody of a composition after only 4–5 listens. A teacher will usually break the composition down into sections and get the student to repeat each section until they are able to reproduce them accurately, before reassembling the sections until the whole *sthāyī* and *antarā* can be sung entirely. This strategy undoubtedly develops a student’s musical ear for the intricate details and nuances of a melody, and teachers are careful to ensure that students pick up these melodies and sing them accurately and with precise intonation. The extent to which a teacher’s rendering of a *bandiś* is consistent from lesson to lesson can be an issue for learners. Some teachers insist that they always sing the *bandiś* in the same way, in opposition to their students’ assertions (Morris, 2005, p. 61).

These discrepancies could be attributed to a natural part of the creative process, it is not uncommon for performers to forget lyrics or small parts of a composition and to consult other performers or recorded performances of the *bandiś* for clarification (see example in section 4.2). Given that musicians are used to constructing phrases to fill gaps or joining pieces of melodic material together to create improvisations, it is not too much of a leap to consider that this may also occur in the remembering of a *bandiś*. Morris provides an excerpt from an interview with Sharadchandra Arolkar in which he asserts “A *ḵẖayāal* song is not a frozen thing; it’s a fluid sculpture…The substance should be there - the expression, the meaning - but [it’s] not like tracing. You have to create, not to trace by memory” (Morris, 2005, p. 67). This example demonstrates that there are two ways in which learning compositions helps develop students’ improvisation strategies. Firstly, it improves their memories for complex melodic material and their competence in singing ornaments. Secondly, it increases a student’s understanding of the *rāg* and the quantity of improvisational objects that they can draw on to manifest the *rāg* in performance. Having a large stock of *bandiśes* can be seen as an alternative strategy to learning the grammar of the *rāg* through the memorisation of the ascending and descending grammar of each *rāg* where certain notes may be omitted or emphasised in ascent or descent.

In pedagogical terms, the impact of the development of *sargam* for use by teachers and students is highly significant to all sections of performance, including the *bandiś*. By creating strong associations between the name of the note and the pitch of the note, teachers are not only developing singers who can sight read the basic shape of a *bandiś* from notation (as in Kodály pedagogy), but they are also building the foundations for pupils who can perform their own precomposed or improvised *ālāps* and *tāns* sung in *sargam*. For pupils who struggle to pick up phrases from their teacher by ear, compositions notated in this form provide important visual cues for the musical material that comes next.

#### 3.4.7 Improvisatory processes: successive variation strategies, compositional strategies, transposition strategies and subverting the audience’s expectations

The idea of syntax of musical units supports a generative view of improvisation in that a performer can string together musical units in novel ways if they conform to the conventional syntax of the musical style. Implicit understanding of melodic syntax is also vital if a listener is to enjoy a performance of Indian music, and if a performer is to use that understanding to reinforce or subvert expectations of how a particular phrase will progress. A musical event that signals that a phrase will be ending warns the listener to expect closure. And, in the same way, musical units can also fulfil a beginning or middle function. Explains that, when formulas are repeated according to the ‘successive variation strategy’ that she has defined, their function is to remind the listener of their previous occurrences, creating a sense of familiarity with the material that can then be varied in order to add interest and a sense of development to the performance. The formulas themselves delineate the structure of a particular section of the music and create a sense of musical syntax.

Compositional strategies of the type identified by Alaghband-Zadeh (2013) and Clayton (2000) have been identified in the literature that analyses performances, but the strategies used to teach them are not fully understood. There is a greater representation of the strategies used by teachers to develop the process of successive variation strategies and the literature also highlights the prevalence of transposition strategies (Manuel, 1989; Nooshin and Widdess, 2006; Alaghband-Zadeh, 2013). These are often presented to emphasise the role that listening to professional performances by *gurus* and others must play in learning phrases that can be appropriately transposed into different *rāgs*. It is likely that this strategy extends to the way in which students learn the process of subverting the audience’s expectations, for example by extending a phrase past the conventional closing figure.

#### 3.4.8 Improvisatory processes: *khaṇḍadmēru*

At the stage of learning to sing *palṭās,* a process called *khaṇḍamēru* is often introduced to students. This involves a mathematical process of singing all the possible combinations of notes in groups (usually 3 or 4). Table 1 demonstrates the permutations for the groups of three notes. Degrees of the scale have been given as numbers as well as the first letter of the *sargam* syllable (S = *sa*, R = *re*, G = *ga*, M = *ma*, P = pa, D = dha, N = ni). Students will sing these phrases column by column.

**Table 1:** Example of permutations of 3 notes when *khaṇḍamēru* process is applied.

		In the *khaṇḍamēru* process, students will sing each of these phrases column by column starting in the top left							
		1 Permutations of SRG	2 Permutations of RGM	3 Permutations of GMP	4 Permutations of MPD	5 Permutations of PDN	6 Permutations of DNṠ	7 Permutations of NṠṘ	8 Permutations of ṠṘĠ
Each row shows a different permutation of the three degrees of the scale specified by each column	a	SRG 123	RGM 234	GMP 345	MPD 456	PDN 567	DNṠ 67 $\dot{1}$	NSṘ 7 $\dot{1}\dot{2}$	ṠṘĠ $\dot{1}\dot{2}\dot{3}$
	b	RSG 213	GRM 324	MGP 435	PMD 546	DPN	NDṠ 76 $\dot{1}$	ṠNR**˙** $\dot{1}$ 7 $\dot{2}$	ṘṠĠ $\dot{2}\dot{1}\dot{3}$
	c	SGR 132	RMG 243	GPM	MDP 465	PND 576	DṠN 6 $\dot{1}$ 7	NṘṠ $\dot{2}\dot{1}$	ṠĠṘ $\dot{1}\dot{3}\dot{2}$
	d	GSR 312	MRG 423	PGM	DMP 645	NPD	ṠDN $\dot{1}67$	ṘNṠ $\dot{2}7\dot{1}$	ĠṠṘ $\dot{3}\dot{1}\dot{2}$
	e	RGS 231	GMR 342	MPG	PDM 564	DNP	NṠD $7\dot{1}6$	ṠṘN $\dot{1}\dot{2}7$	ṘĠṠ $\dot{2}\dot{3}\dot{1}$
	f	GRS 321	MGR 432	PMG	DPM 654	NDP	ṠND $\dot{1}76$	ṘṠN $\dot{2}\dot{1}7$	ĠṘṠ $\dot{3}\dot{2}\dot{1}$

Once students can sing this combination of notes fluently, teachers will add challenges to apply ornamentation to the permutations of notes. These units of three or four notes can also be combined vertically to produce longer patterns. Tables 2, 3 demonstrate two different formulas by which the *khaṇḍamēru* passages are commonly combined.

**Table 2:** Example of *khaṉḏamēru* patterns combined horizontally.

	Passages sung by students which combine *khaṉḏamēru* phrases					
	1st sung phrase	2nd sung phrase	3rd sung phrase	4th sung phrase	5th sung phrase	6th sung phrase
*sargam*	SRG, RGM	RSG, GRM	SGR, RMG	GSR, MRG	RGS, GMR	GRS, MGR
Degrees of the scale	123, 234	213, 324	132, 243	312, 423	231, 342	321, 432
Formula using figures from Table 1	1a + 2a	1b + 2b	1c + 2c	1d + 2d	1e + 2e	1f + 2f

Or combined vertically:

**Table 3:** Example of *khaṇḍamēru* patterns combined vertically.

	Passages sung by students which combine *khaṇḍamēru* phrases							
	1st sung phrase	2nd sung phrase	3rd sung phrase	4th sung phrase	5th sung phrase	6th sung phrase	7th sung phrase	8th sung phrase
*sargam*	SRG RSG	RGM GRM	GMP MGP	MPD PMD	PDN DPN	DNṠ NDṠ	NṠṘ ṠNṘ	ṠṘĠ ṘṠĠ
Degrees of the scale	123 213	234 324	345 435	456 546	567 657	67 $\dot{1}$ 76 $\dot{1}$	7 $\dot{1}\dot{2}$ $\dot{1}7\dot{2}$	$\dot{1}\dot{2}\dot{3}$ $\dot{2}\dot{1}\dot{3}$
Formula using figures from Table 1	1a + 1b	2a + 2b	3a + 3b	4a + 4b	5a + 5b	6a + 6b	7a + 7b	8a + 8b

This process of developing students’ mental agility for all possible combinations of notes supports the development of both memory for precomposed *tāns* and the ability to improvise melodic material in *sargam*. By strengthening the indexical link between the production of the note and the phoneme for notes that do not just follow in step but also contain wider intervals, the *tāns* that students are able to memorise will include greater variety as time goes on. Learning by rote is the pedagogical strategy by which the process of *khaṇḍamēru* is learned, but when deployed in the context of a performance can be incorporated creatively within an improvisation.

#### 3.4.9 Improvisatory processes: *behlāvā*

Another technique used by teachers to develop their students’ improvisatory processes is *behlāvā*: decorating the words of the composition with notes that are different from the ones formerly used. By repeating the same phrase, several times, the potential for variation in a single phrase and with a limited range of notes explored. This again demonstrates how teachers set parameters for improvisation in order to support students’ creative thinking. McIntosh (1993) explains that another restriction imposed on *behlāvā* is that the improvised phrase should only take one or two metric cycles before returning to the composition, in order to prevent *behlāvā* from losing its artistic effect. *Behlāvā* is an improvisational strategy that lends itself to being taught within the GSP, because it is an exploratory process that can be engaged in during *tālīm* by both *guru* and *śiṣya* concurrently.

#### 3.4.10 Improvisatory processes: intensification strategies

Intensification is a concept referenced in much of the literature as the overarching process by which improvisation occurs in Indian classical music (Clayton, 2000; Henry, 2002; Nooshin and Widdess, 2006; Zadeh, 2012). Clayton considers that the process of intensification during a performance occurs across a number of musical continua—tempo, register and complexity (Clayton, 2000). Nooshin and Widdess (2006) agree that rhythmic intensification through the gradual increase of tempo or rhythmic density is a fundamental process of development in improvisation. Building on the work of Kramer (1988), Clayton considers that the structural aspects of intensification are linear (deductive and sequential), but that, rather than leading to a precise musical climax when the *upaj* is seen as complete (a teleological strategy), it instead intensifies cumulatively, up until the point at which the limit of a performer’s technical ability has been reached, the point at which the time allowed for their performance is almost up (and in some cases exceeded) or until a performer becomes bored with the process. This suggests that the overall impression gained by the listener is of a non-linear, holistic, and continuous process. Clayton links this improvisatory process to cultural ideologies, asserting that, whilst there is a Western tendency to theorise and attempt to demonstrate logical organisation and coherence within the cultural phenomenon of music, Indian music theorists are much more likely to assert music’s attributes as a state of being. “Thus, a *rāg* simply *is*: the performer’s task is to bring the *rāg* to the listeners’ consciousness and allow us to focus our attention on the *rāg’s* qualities” (Clayton, 2000, p. 26). The implication of this for learners is that it highlights the teacher’s role in pitching their modelling of intensification at an appropriate level for a student to be able to replicate. It also supports the strategy of seeing *rāg* as a more complex musical entity than can be grasped by memorisation of the ascending and descending phrases of the *rāg*. For students, learning characteristic phrases sung by their teacher therefore helps to develop the precise dialectic and idiolectic sensibilities needed to construct fluent performances.

### 3.5 A note on the transcriptions

This next section of the article uses numerical notation in place of *sargam* (syllables used to name the notes of a scale in a *rāg*); hence ‘*sa re ga ma pa dha ni*’ is written ‘1 2 3 4 5 6 7’. Presenting degrees of the scale as numbers helps to demonstrate patterns more clearly to readers less familiar with the *sargam* system. Upper and lower octaves (*saptaks*) are denoted by a dot above or below the number (for example, 7̣, $\dot{1}$ ). If the scale has been altered from the *bilāval* scale/*ṭhāt* (or Ionian mode) then this is denoted by a line above or below the number to demonstrate if the note (*svar*) has been raised (*tīvră*) or flattened (*komal*). Hence the ascending *bhairav* scale*/ṭhāṭ* (double harmonic major scale) is notated ‘1 2 3 $\underline{4}$ 5 $\underline{6}$ 7 $\dot{1}$ ’ and the *kalyāṇ ṭhāṭ* (Lydian mode) is notated ‘1 2 3 $\bar{4}$ 5 6 7 $\dot{1}$ ’. Where there is a rest, or a note has been held for twice as long in the pattern, this has been transcribed as –.

## 4 Illustrative examples

The differences between teaching in the GSP and in music schools are identified in section 3.1; however, the way these differences present themselves on a lesson-to-lesson basis in these specific cases is identified through analysis of the video data from observations. Learners are referred to as *śiṣyas* in the GSP context and students in the music school context. This section begins with an analysis of the pedagogical strategies used in two recorded observations: one from a music school and one from the GSP. Following this, a thematic discussion presents the implications for understanding the pedagogical strategies used to develop improvisation skills in NICM. Illustrative example 1 takes place in a music school in Varanasi with a female teacher and eight beginner students. The styles they are learning are *ḵẖayāl* and *ṭhumrī* and they cover *rāg yaman kalyāṇ*, *malkauns* and *bilāval* during the lesson. Illustrative example 2 also takes place in Varanasi at the home of a male *guru* with four experienced *śiṣyas* aged 14–17. The styles they are learning are also *ḵẖayāl* and *ṭhumrī* and they cover *rāg yaman kalyāṇ* during the *tālīm*.

### 4.1 Music school teaching

This lesson took place on the evening of March 20, 2018, at a music school in Varanasi. Starting at 6 pm and lasting just over an hour as the sky transitioned from daylight to darkness. The atmosphere was relaxed, with some chatter and laughter among the teacher, students, and parents, yet there was also a clear emphasis on maintaining focus. This session involved only women and children; unlike other lessons I had observed in the music school where the chairman was also present. The class consisted of five girls and three boys aged 7–11, with two mothers sitting at the back, occasionally chatting with the teacher. As a customary practice, students left their shoes in the exterior corridor, touched the teacher’s feet upon entering, and had their heads touched by the teacher before sitting. They sat cross-legged in a semicircle with the teacher at the front, boys on one side and girls on the other, although it was unclear if this seating arrangement was enforced or habitual.

The lesson began with the teacher singing a long, slow “sa,” joined by the students, followed by “pa.” After several repetitions, the students were split into groups of two and three to sing 1,234,567 $\dot{1}\dot{1}7$ 654321 in *sargam* and *ākār* (singing pitches to the syllable ‘aa’), with a focus on intonation. Older and more confident students demonstrated better intonation and smoother transitions, as well as greater ease with the faster *palṭās* compared to younger students, who struggled with the phonemes at higher speeds. The pattern 1122334455, 123323443455 (familiar to the group) was sung in *sargam* and *ākār*, with the teacher only needing to start each variation for the students to anticipate the next sequence. Following this, the pattern of singing *palṭās* in *sargam* and *ākār* is illustrated in Table 4.

**Table 4:** Examples of *palṭās* sung in *sargam* and *ākār* from illustrative example 1 (music school teaching).

	*palṭās* sung by students during illustrative example 1 (presented using degrees of the scale)	
	Ascending phrases of the palṭās sung by students	Descending phrases of the palṭās sung by students
1st palṭā	1233, 2344, 3455, 4566, 5677, 67 $\dot{1}\dot{1}$	$\dot{1}$ 766, 7655, 6544, 5433, 4322, 3211
2nd palṭā	121233, 232344, 343455, 454566, 565677, 6,767 $\dot{1}\dot{1}$	$\dot{1}$ 7 $\dot{1}$ 766, 767655, 656544, 545433, 434322, 323211
3rd palṭā	1-32132132121321, 2-43243243232432, 3-54354354343543, 4-65465465454654, 5-7-6576576565765, 6- $\dot{1}$ 76 $\dot{1}$ 76 $\dot{1}$ 7,676 $\dot{1}$ 76	5-76576576565765, 4-65465465454654, 3-54354354343543, 2-43243243232432, 1-32132132121321
4th palṭā	13, 24, 35, 46, 57, 6 $\dot{1}$	$\dot{1}$ 6, 75, 64, 53, 42, 31

In this lesson, all *palṭās* were sung with *tablā* accompaniment set at 160 *mātrās* (beats) per minute, which is on the cusp between *drut lāya* (fast tempo) and *madhya lāya* (medium tempo), with each *svar* lasting half a *mātrā*. Despite some students struggling with this tempo, the teacher emphasised the importance of mastering these *palṭās* at this speed in both *sargam* and *ākār*. This approach ensures that students internalise these patterns well enough to recite them fluently at any speed during improvisation, representing an efficient drill method. The melodic features of the *palṭās* include repetition and variation, akin to the *tāns* used in performance. Although the *palṭās* are simple and in the *bilāval* scale/*ṭhāṭ* (ionian mode), the strategy of internalising these patterns for future use in improvisation is evident. The ascending and descending phrases of the *palṭās*, confined to a narrow pitch range (no greater than a third), also reflect how *tāns* are constructed in performance.

In this lesson, the teacher uses body language and non-verbal cues to indicate satisfaction with students’ accuracy during *palṭās*. When correcting errors, she would repeat a short segment to improve students’ ability to copy phrases by rote. Although students sing more confidently in unison, this does not necessarily improve their accuracy. The teacher segments challenging *palṭās* for struggling students, modelling an effective practice strategy of breaking down longer phrases into manageable chunks. She speaks very little during the lesson, except for a short section where she tests students’ ability to recognise different *tāl* patterns.

Two-thirds of the way through the lesson, students sing a pre-composed *bhajan* (devotional song) in *rāg yaman kalyāṇ*, set to *rūpak tāl* (a 7 beat cycle). This scale/*ṭhāṭ* differs from the earlier *palṭā* activity. After rehearsing the *bhajan*, students move on to a *choṭā ḵẖayāl*. The teacher begins by asking students to sing the *ārōh* (ascending pattern) and *avarōh* (descending pattern) of *rāg malkauns*. Initially, students struggle with the pitches due to the lingering memory of the *kalyāṇ* scale/*ṭhāṭ* from the *bhajan*. The teacher then sings the *ārōh* and *avarōh* slowly with correct pitches (1-3̲-4-6̲-7̲- $\dot{1}$ - $\dot{1}$ -7̲-6̲-4-3̲-4-3̲-1), which students repeat. Once this scale/*ṭhāṭ* has been established, students repeat the *ālāp* phrases shown in Table 5.

**Table 5:** Examples of *ālāp* phrases sung by the teacher and repeated by the students in illustrative example 1 (music school teaching).

	*ālāp* phrases sung by students during illustrative example 1 (presented using degrees of the scale)		
1st ālāp phrase	6̱̣-7̲̣-1-4---	6th ālāp phrase	4-6͟-7̲-7̲-6̲-
2nd ālāp phrase	4----3̲-	7th ālāp phrase	3̲-46̲-7̲ $\dot{1}$ ----
3rd ālāp phrase	3̲-4-3̲-1-	8th ālāp phrase	$\dot{3}$ _- $\dot{1}$ -7̲- $\dot{1}$ -6̲-
4th ālāp phrase	6̲̣-7̲̣-1-4---4----3̲-3̲-4-3̲-1---	9th ālāp phrase	7̲-6̲-7̲-7̲-6̲-4---
5th ālāp phrase.	1-3̲-4-6̲-	10th ālāp phrase	3̲-4-6̲-3̲-4-3̲-7̲̣-6̲̣-1---

This sequence demonstrates how the teacher develops students’ memories for characteristic phrases that follow the grammar of the *rāg* (unlike *palṭās*). For instance, phrase 4 is a combination of phrases 1, 2, and 3, effectively establishing both memory for phrases and the technique of stringing phrases together incrementally. Key features of the *ālāp* phrases include mostly stepwise motion and small jumps (e.g., intervals of a third and fourth in phrase 10) that involve ornamentation to create a more stepwise route through the *ālāp* phrases. The pitch ranges are characteristic of a *ḵẖayāl ālāp*, starting low and expanding upwards (up to $\dot{3}$ ) before descending back to 1.

Following a long-held 1 at the end of phrase 10, the class transitions to singing the *bandiś* of the *choṭā ḵẖayāl*. The teacher adds a *tihāi* based on the first word of the *sthāyī* for students to follow, exemplifying how traditionally improvisatory aspects are composed by the teacher and memorised by students in a music school context. Notably, there is no section for *vistār* in this *choṭā ḵẖayāl*, and students are not expected to copy the improvisatory phrases demonstrated by their teacher, likely due to their current skill level rather than their age.

### 4.2 Guru-śiṣya paramparā tālīm

This episode of *tālīm*, conducted on 13th January 2018 at 8:00 pm, takes place in the *guru’s* home. The four male *śiṣyas* are dressed in thick western attire due to the cold and were seated on a raised dais facing their *guru*. The itabla app provided drone and *tablā* accompaniment during the lesson, which followed the structure of a full performance of *rāg yaman kalyāṇ*. The session began with the *ālāp* section, where *śiṣyas* repeated phrases sung by the *guru* in *ākār* as a group. The *guru* repeated intricate phrases with verbal explanations to highlight specific expressive features, sometimes singing alongside the *śiṣyas* for support. Head gestures were used by both the *guru* and *śiṣyas* to indicate the accuracy and mastery of the phrases.

Following the *ālāp*, the lesson focused on perfecting a very slow precomposed *bandiś* in *vilambit ektāl* (a slow, 12 beat cycle). The *guru* began by singing the first phrase of the *bandiś* without *tablā* accompaniment, which the *śiṣyas* copied as a group. After starting the itabla app, the *guru* had to reset the *tablā* accompaniment due to a rhythmic issue. He consulted a book of notated *bandiśes* to verify the correct *tāl*, then restarted the *tablā*, and the group continued singing the *sthāyī* in unison. The use of notated *bandiśes*, both printed and handwritten, highlights a distinctive feature of this *tālīm*. While some *gurus* rely solely on memory, in this instance, *śiṣyas* had access to printed materials to aid their learning.

Once the *ālāp* section concluded, the *guru* and *śiṣyas* rehearsed aspects of *vistār*. The *guru* started by singing lengthy phrases that *śiṣyas* struggled to remember, necessitating reminders and breaking the phrases into smaller chunks. Sometimes, the *guru* sang along with the students for support, while at other times he listened and corrected them by interjecting. In the second part of the *vistār* section of the *baṛā ḵẖayāl*, individual *śiṣyas* sang their own improvisations interspersed with the first phrase of the *sthāyī*. The *guru* listened and provided musical suggestions, modelling and explaining as needed. When one *śiṣya* missed the correct beat of the *tāl* to start the *sthāyī*, the *guru* demonstrated an alternative phrase before moving on to the next student. The *śiṣyas* recorded their *tālīm* on their phones, allowing them to review the improvisations later. Each student incorporated aspects of the *guru’s* phrases into their improvisation, and all listened intently to each other.

In the next section of the *baṛā ḵẖayāl vistār*, the *guru* modelled variations on the *sthāyī*, which *śiṣyas* repeated either as a group or individually. Sometimes, the *guru* focused on a single word of the lyrics, constructing phrases for individual *śiṣyas* to repeat. The individual sections were interspersed with the *sthāyī*, and some phrases were repeated as a group. Those not singing paid close attention to each other’s phrases, sometimes shaping the air with hand gestures to reflect the melodies. As the lesson developed, the pitches expanded into the upper register, continuing into the higher octave and including the characteristic long-held high *sa*. The *śiṣyas* copied their *guru’s* phrases in unison using the text of the first line of the *antarā*. The format continued with the group singing the *sthāyī* of the *baṛā ḵẖayāl bandiś* in *ākār*, interspersed with variations on the *antarā*, and finished with the first line of the *sthāyī*.

The next section of the *tālīm* focused on *sargam* and *bol tāns* as part of the *baṛā ḵẖayāl*. The *tāns* shown in Table 6 are demonstrated by the *guru* and copied in unison by all *śiṣyas*. Later *tāns* in the sequence are focused on individual *śiṣyas*, but for the purpose of this analysis, that detail has been omitted from the transcription below.

**Table 6:** *bol tāns* sung as part of the *baṛā ḵẖayāl* from illustrative example 2 (GSP *tālīm*).

	*tāns* sung by students during illustrative example 2 (presented using degrees of the scale)		
1st tān	3-23217̣6̣7̣23-	13th tān	54̅3234̅67 $\dot{1}7\dot{1}7$ 654̅64̅534̅2321
2nd tān	7̣237̣27̣323217̣23---	14th tān	54̅3234̅67 $\dot{1}7\dot{1}7$ 654̅64̅534̅23217̣6̣7̣234̅3
3rd tān	7̣237̣27̣32321---	15th tān	$\dot{2}$ 76765
4th tān	4̅4̅34̅32, 332321, 7̣6̣7̣234̅3-----	16th tān	5767, 5645, 34̅2321----
5th tān	7̣234̅3-	17th tān	333, 777, $\dot{3}\dot{3}\dot{3}$ , 7 $\dot{2}\dot{1}$ 7, 654̅654̅34̅234̅324̅321
6th tān	4̅4̅34̅32, 332321, 7̣6̣7̣234̅3-----	18th tān	7̣234̅32, 7̣234̅54̅32, 34̅6777
7th tān	7̣234̅54̅32, 34̅54̅32, 34̅654̅3234̅3-----	19th tān	4̅6764̅64̅7655654̅34̅2321
8th tān	7̣234̅54̅32, 7̣234̅654̅32, 7̣234̅54̅3234̅3---	20th tān	154̅534̅2321 (x3)
9th tān	34̅7654̅32321	21st tān	76756564̅34̅321 (x2)
10th tān	54̅3234̅6765764̅64̅534̅2321	22nd tān	7̣234̅3-, 76756564̅34̅321
11th tān	4̅67464767654̅654̅2323---	23rd tān	333, 777, $\dot{3}\dot{3}\dot{3}$ , 7 $\dot{2}\dot{1}$ 7, 654̅654̅34̅234̅324̅321
12th tān	34̅323-23217̣6̣7̣23---	24th tān	7̣234̅54̅, 234̅567, $\dot{3}\dot{2}\dot{1}\dot{2}\dot{1}7\dot{2}\dot{1}$ 7654̅, 654̅34̅2321

In order to demonstrate the number of times that key fragments are repeated within the improvised *bol tāns*, Table 7 records the number of repetitions of key phrases within illustrative example 2. This demonstrates how melodic material is being repeated in the *bol tāns* of both the *baṛā* and *choṭā ḵẖayāl*.

**Table 7:** The number of times that common note combinations are repeated during two sections illustrative example 2 (GSP *tālīm*).

	Common note combinations in the *bol tāns*	Number of times phrase is repeated in the *bol tāns* of the *baṛā ḵẖayāl*	Number of times phrase is repeated in the *bol tāns* of the *choṭā ḵẖayāl*	Total
Common note combination 1	323217̣6̣7̣23	4	10	14
Common note combination 2a and 2b	64̅534̅2321 and 654̅34̅2321	6 (4 and 2)	0	6 (4 and 2)
Common note combination 3a and 3b	3217654̅32 and $\dot{3}\dot{2}\dot{1}$ 7654̅32	0	12 (8 and 4)	12 (8 and 4)
Common note combination 4a, 4b and 4c	7̣234̅3 and 7̣234̅5 and 72354̅	11 (6 and 5 and 0)	8 (3 and 2 and 3)	19 (9 and 7 and 3)
Common note combination 5a and b	34̅2321 and 32321	14 (7 and 7)	13 (1 and 12)	27 (8 and 19)
Common note combination 6 a and b	54̅3234̅ and 234̅67	15 (11 and 4)	10 (6 and 4)	25 (17 and 8)
Common note combination 7a, b and c	654̅654̅ and 654664 and 654645	6 (6 and 0 and 0)	6 (3 and 2 and 1)	12 (9 and 2 and 1)
Common note combination 8a, b, c and d	654̅23 or 654̅32 or 64̅534̅ or 654̅34̅	12 (1 and 3 and 4 and 4)	23 (0 and 17 and 5 and 1)	35 (1 and 20 and 9 and 5)

In this example, the *guru* used a method of constructing improvisations with increasing intensity that closely resembled aspects of a *ḵẖayāl* performance. This approach involved the repetition of small melodic fragments and entire *tāns*, creating familiar patterns. Key techniques included reusing previously sung *tāns* within longer *tāns* and employing specific phrases for syntactic functions, such as ending or beginning motifs. For instance, *baṛā ḵẖayāl tān* 1 (323217̣6̣7̣23) appeared within *tān* 4, 6 and 12, while *tān* 2 (7̣237̣27̣32321) reappeared in *tān* 3. *Tān* 4 (4̅4̅34̅32, 332321, 7̣6̣7̣234̅3-----) is repeated within *tān* 6. Furthermore, certain phrases seem to fulfil specific syntactic functions for example, *tān* 5 (7̣234̅3-) is repeated within *tān* 4, 6, 14, 18 and 22 as a motif by which to end the phrase. As well as *tāns* that end idiomatically, there are motifs that are frequently used at the beginnings too. For instance, *tān* 10, 13 and 14 start in similar ways (although they are not identical) but they do end in the same way (64̅534̅2321). *Tān* 11 and 19 start in the same way (4̅6764̅64̅76) as do *tān* 17 and 23 (333,777, $\dot{3}\dot{3}\dot{3}$ ).

Variation was another strategy used, for example, *tān* 7 and 8 were similar but not identical, with *tān* 8 developing the phrase from *tān* 7 while maintaining the same ending. This technique of combining or altering phrases allows for expressive possibilities and increased intensity in the improvisation. Advanced *śiṣyas* apply these ideas in different contexts, such as within the *bol tāns* of the *choṭā ḵẖayāl*. The *tāns* maintained a consistent fast speed and showed little rhythmic variation, indicating that the *śiṣyas* had mastered slower speeds and were now developing their skills at a higher tempo. The pitch range expanded throughout the sequence, mirroring the *ālāp* and intensifying the improvisation. The *tāns* typically ended on 1, 5, or 3, with only one exception (18), which upon closer inspection, was part of a longer phrase ending on 1.

Following the *sargam tāns*, the work was consolidated through the *ākār tāns*, reinforcing the strategies and techniques learned during the session. This methodical approach highlighted the importance of both repetition and variation in developing *śiṣyas’* improvisational skills.

## 5 Results and discussion

The illustrative examples from music school and GSP highlight strategies for developing improvisation skills in students at both early and advanced stages of training. In music schools, lessons for younger students are divided into smaller sections, allowing frequent repetition of characteristic *rāg* phrases to aid long-term memorisation. As described by Sanyal and Widdess (2004) and Alaghband-Zadeh (2013), lessons are structured to mirror performances, helping students form expectations and schemas which comply with conventions such as pitch ranges and phrase variation. High expectations for focus and respect are common across teaching settings, whether in music schools or in the GSP in line with the conventions observed by Kippen (2008, p. 131). Students are expected to listen silently and learn from others and their teacher, without asking questions. If students struggle, teachers use repetition, segment material or adjust the pace.

More experienced students improvise their own *tāns*, balancing repetition and variation to avoid unstructured phrases, a strategy that is discussed by teachers and recorded in an archive recording (Dey, 2009) but does not appear in the literature. Strategies for *tāns* include maintaining pitch range parameters, using melodic and rhythmic variations, and rearranging note combinations like *khaṉḏamēru* (Qureshi, 2007, p. 92). Imitation of modelled phrases is the predominant teaching strategy (Mirza Maqsud Ali cited in Qureshi, 2007, p. 190). Even advanced students rarely demonstrate spontaneous improvisation, focusing instead on memorising long phrases. This practice helps them eventually create their own phrases. Advanced students develop the ability to improvise through familiarity with the repertoire and understanding the construction principles of patterns, rather than just memorising note sequences (Powers, 1980; Neuman, 2012; McNeil, 2017). Teachers demonstrate the expansion of phrases in *ālāp* and *tāns*, showing students how to develop and extend phrases. While *palṭās* act as maps of tonal space (Magriel, 1999), the *ālāp* and *tāns* enable performers to explore both familiar and new pathways through the tonal space, ultimately guiding students towards independent improvisation.

### 5.1 The foundational skills of simple scale exercises

The simple scale exercises observed in music schools serve multiple functions in musical training, such as developing students’ voice control, precise intonation, breath control, and secure positioning of *s*var*s* in memory. These exercises help create an indexical link between the notes and their names within the *sargam* system and support the development of simple *alaṅkārs* (ornaments), where repeating notes often leads to the natural addition of *gamak* to embellish each note (Magriel, 2002). Intonation is particularly challenging for young vocal music students, so secure positioning of *s*var*s* within a child’s comfortable singing range is emphasised from the first lesson. This involves drilling *palṭās* alongside singing long, slow notes while focusing on listening to the *tānpūrā* drone or *śruti box* to check intonation. *Palṭās* are efficient in training pitch control due to the wide range of note permutations within a scale. The repetitive nature of these drills allows teachers to assess whether students have internalised the pitches and can accurately reproduce them in any order, even when their minds wander during the exercises.

### 5.2 Transition from scale exercise to *palṭās*

Once students/*śiṣyas* have mastered simple scale exercises, the introduction of *palṭās* further develops their voice control, internalisation of complex pitch patterns, ability to transpose pitch patterns, and cognitive mapping of scale structures, independently of *rāg*. *Palṭās* are initially sung slowly in *sargam*, then in *ākār*, before speeding up, showing the precise strategies used for internalising these patterns over time. By incorporating *palṭās* with different leaps over a sequence of lessons, and expecting practice between lessons, students develop precise intonation of various intervals. Once the routes to *svars* become automatic, students gain ease in sequencing and varying melodic material, a crucial skill for fluent improvisation. As students internalise numerous patterns, they can recall them and produce or link specific musical phrases. These patterns, useful for starting, linking, or ending phrases, are vital tools for memorisation in preparation for performance (Magriel, 1999).

In a *ḵẖayāl* performance, the final section which incorporates *tāns*, relies heavily on a performer’s ability to improvise. Teachers use various strategies to develop students’ skills to compose and improvise *tāns* that are complex yet conform to the *rāg’s* grammar. The basic skills for performing *tāns*, both compositional and technical, are taught through *palṭās*. In advanced training, students learn to memorise, compose, and eventually improvise *tāns* that retain many features of the earlier *palṭās* and reflect the *rāg’s* grammar. This progression illustrates how the strategy evolves from a pedagogical drill to a key feature of improvisation.

### 5.3 Memorising *bandiśes* supports improvisational competence

The *bandiś* material is crucial for students to internalise key phrases of a particular *rāg*, whilst capturing the expressive nuances of melodic and rhythmic phrases. Teachers and *gurus* emphasise the accurate rendering of the *bandiś*, including ornamentation and pronunciation, ensuring precise knowledge construction (Morris, 2005). While variation in performance is valued, the faithful reproduction of *bandiśes* as taught by the *guru* is prioritised. This reflects the respect for *gurus* and the cultural values associated with musical learning.

Students develop schema-based expectations for the pitch direction of *bandiśes* through enculturation. They learn early that the *sthāyī* melody moves in the lower tetrachord of the middle and lower octaves, while the *antarā* rises to the upper tetrachord and upper octave (Widdess, 1981; Clayton, 2000), aiding efficient learning. Although classificatory knowledge of the *rāg* is less prioritised in the GSP than in music schools, modern GSP teaching often includes theoretical aspects. *Gurus* may publish and print *bandiśes* they learned and share performances online, blending traditional aural learning with modern resources.

Learning *bandiśes* aurally develops associative memory chains (Farrell, 2012), while written notation aids content-addressable memory (Chaffin et al., 2016). Internalising the structure of the *bandiś* is essential for improvisation, with a rule-governed approach emphasised for younger students. Teachers in the illustrative examples adapted their teaching in real time, checking individual students’ progress. Over time, performers can develop an extensive repertoire of 400–1,500 *bandiśes* (Morris, 2005). This extensive repertoire offers an alternative to explicitly learning *rāg* grammar, potentially influencing the speed at which improvisational skills develop.

### 5.4 The importance of practising *ālāps* of developing length and complexity

The internalisation of *rāg* conventions extends beyond the memorisation of bandiśes to include *ālāp* phrases learned over time. These phrases help students grasp *rāg* grammar, incorporating the expansion of phrases, range, and development of musical ideas. Performance conventions of the *ālāp* section vary, with some *gurus* emphasising analytical skills while others do not. Teachers often retain *sargam* syllables in lessons to highlight these patterns and develop students’ analytical abilities.

Beginners are often encouraged to improvise within a limited note range in individual *tālīm*, more so than in group settings. *Gurus* vary in their approach: some teach precomposed *ālāp* phrases for memorisation, while others encourage constructing and improvising phrases from the start. The reordering of phrases, as described by Magriel (2002), applies to both *ālāp* and *tāns*. Expectations may differ based on students; for instance, foreigners might memorise fixed phrases while enculturated learners improvise from the beginning. This aligns with observations by Nooshin and Widdess (2006) suggesting that years of practice are needed before going beyond memorised material. Teachers assess students’ abilities to memorise and repeat *ālāp* phrases, organising groups by skill level rather than age. Differentiating the length and complexity of phrases according to each student’s stage of learning is a key strategy in both music schools and GSP.

### 5.5 Memorisation of *palṭās* supports the creation of *tāns*

The ability of students to repeat phrases modelled by their teacher enhances their capacity for rapid memorisation and instant replication of complex pitch sequences in *rāg* and *tāl*, known as *tāns*. This skill, along with the development of musical ideas and pitch range, is supported by memorising *palṭās*. The principles of repetition, sequence, and variation in *palṭās* also apply to *tāns*. Advanced techniques in *palṭās*, such as combining shorter phrases or repeating steps, help students extend and develop phrases within a metrical structure, aiding performance skills. The potential cognitive differences in enculturated learners, which might necessitate a less structured approach, present an interesting area for further study.

### 5.6 The impact of lesson structure mirroring a performance

The GSP is regarded as the gold standard for musical transmission in NICM. However, both music school settings and GSP share common teaching features, particularly following the *dhrupad* or *ḵẖayāl* structure by starting with *ālāp* or *bandiś*. This sequence models the concept of intensification, a key process in NICM, every time students engage in *tālīm*. Over time, this consistent strategy builds students’ confidence in their performance trajectory, allowing them to focus on intensification details across pitch, rhythm, tempo, melodic complexity, and emotion. Learning various improvisatory techniques enhances students’ abilities to create increasing intensity in performance. The structure of *tālīm* varies with student levels; advanced learners may focus on one *rāg* in detail, while younger or less advanced learners might study a variety of *rāgs*, including film songs and *bhajans* in music schools.

Magriel’s (2002) analysis of young singers’ performances revealed that they used the same melodic material but varied the frequency and sequence of phrase repetitions. This reordering of memorised material is a crucial stage of improvisational competence, allowing students to experiment with intensification using familiar patterns. For example, in illustrative example 1 students initially struggled with the *ārōh* and *avarōh* of *malkauns*, indicating they might find independent improvisation challenging. This mirrors performance structures for pedagogical reasons. The teacher likely uses familiar *bandiś* to establish *s*var*s*, suggesting that increased familiarity with the repertoire will correct students’ mistakes and improve their improvisational skills.

### 5.7 Prevalence of improvisation in lessons/*tālīm*

An important finding from the illustrative examples is that students, particularly those in music schools, are rarely asked to improvise in lessons/*tālīm*. Instead, they focus on memorising and instantly replicating their teachers’ materials. These materials, composed and improvised by the teachers, exemplify the compositional methods and skills needed for improvisation rather than requiring students to construct novel phrases spontaneously. However, teachers’ strategies might be more subtle than they appear. When students’ replications are not exact due to memory limitations, they are indirectly encouraged to recompose their teachers’ phrases spontaneously. This approach, which may seem like rote learning, fosters students’ creative problem-solving abilities in the moment. Teachers may accept inexact replications if they retain the original’s overall intentions and correctness in *rāg* and *tāl*, supporting this assertion. NICM literature supports the idea that memory recall is a creative process, with memories modified and restructured upon recall and influenced by pre-existing schemas that shape expectations. In the observed situations, very advanced students might start creating their own *tāns* in class or independently, though this is primarily a feature of GSP *tālīm*.

## 6 Conclusion

This article set out to identify what the improvisatory objects and processes being used in the training and performance of NICM are through the analysis of qualitative data, generated through video supported observations and fieldnotes. Through this process, seven themes have been identified:

1. The foundational skills of simple scale exercises
2. The transition from learning simple scale exercises to learning *palṭās*
3. The supportive strategy of learning many *bandiśes*
4. Developing the length and complexity of *ālāps*
5. Memorising *palṭās* supports the ability to construct *tāns*
6. Lesson structures mirror performance structures
7. Memorisation is a precursor to improvisation

This article also highlights several pedagogical strategies in NICM that warrant further exploration, particularly the relationship between skills needed for embellishing fixed compositions (*tāns*) and those for improvising non-fixed sections (*ālāp*). Further study is needed to bridge the gap between ethnomusicologists and psychologists in defining music perception and cognition processes in oral traditions. Interdisciplinary work could enhance our understanding of musical development in these contexts. The research also suggests investigating the role of non-verbal gestures in *dhrupad*, *ṭhumrī*, and *ḵẖayāl* that may support learning and performance.

The cognitive differences between learners enculturated in NICM and those from other musical traditions are likely significant. This research has implications for teachers across various musical styles and traditions. For instance, the lack of performance anxiety observed by Qureshi (2007, p. 292) in NICM students, despite the challenging repertoire and high expectations, suggests that a schema-based pedagogy could be beneficial for musicians in all styles. Such a pedagogy could help students from different disciplines develop confidence in their improvisational abilities, addressing an issue common in classical traditions where improvisation is not prioritised.

## Data Availability

The datasets presented in this article are not readily available because I do not have written permission to share videos of children and young people beyond the scope of the illustrative examples used in my PhD thesis. Requests to access the datasets should be directed to emily.sayers@canterbury.ac.uk.

## Appendix A: Glossary of key terms

**Table:**

*Hindī*/*Urdū* terms	Definition
*ākār*	singing pitches to the syllable ‘aa’
*alaṅkār*	musical ornament
*ālāp*	opening section of a *rāg* in North Indian Classical music, a melodic improvisation that introduces the *rāg*
*andaz*	style/how we express ourselves to the world
*antarā*	second section/line of a song, after the *sthāyī*
*ārōh*	ascending scale of notes in a *rāg*
*avarōh*	descending scale of notes in a *rāg*
*bandiś*	fixed melodic composition in North Indian Classical music, usually for voice but also used for instrument
*baṛā ḵẖayāl*	large (in the context of *baṛā ḵẖayāl* this means long)
*baṛhat*	exposition of the *rāg* through melodic expansion
*behlāvā*	strategy for elaborating on a *bandiś*
*bhairav*	name of a *rāg* and *ṭhāṭ* (sung at dawn) features a flattened 2nd and 6th
*bhajan*	devotional song
*bhāva*	emotional state
*bilāval*	*ṭhāṭ* with intervals similar to the ionian mode
*bol tān*	precomposed or improvised phrases interspersed with sections of the *sthāyī* and *antarā* phrases of the *bandiś*, sung to the words of the *bandiś*
*calan*	the way the *rāg* goes, conventions of the *rāg*
*choṭā ḵẖayāl*	small (in the context of *choṭā ḵẖayāl* this means short)
*deś*	*rāg* in *khamāj ṭhāṭ* (a *ṭhāṭ* similar to the mixolydian mode)
*dhrupad*	oldest surviving form of North Indian classical vocal music
*drut laya*	Fast tempo (between 160 and 320 beats per minute)
*ektāl*	12 beat *tāl*
*gamak*	vocal trill/oscillation
*gharānā*	system of social organisation linking musicians (and often dancers) to a particular artistic style and way of learning
*guru*	teacher, guide, mentor
*gurukul*	residential education establishment following principles of the *guru-śiṣya paramparā*
*guru-śiṣya paramparā*	denotes a lineage of teaching and learning where knowledge is imparted through a relationship between *guru* and *śiṣya*
*hindol*	*rāg* in *kalyān ṭhāṭ* which has similar intervals to the lydian mode
*kalyāṇ*	*ṭhāṭ* with intervals similar to the lydian mode
*ḵẖayāl*	literally ‘imagination’, a modern genre of North Indian Classical Vocal Music (see also *dhrupad*, *ṭhumrī*)
*khaṉḏamēru*	process where 3 or 4 notes patterns are selected, and all the possible permutations of those notes are rendered
*komal*	flattened note
*madhya lāya*	medium tempo (between 80 to 160 beats per minute)
*malkauns*	a pentatonic *rāg* in *bhairavī ṭhāṭ* (where all *svars* are *komal*)
*mukhṛā*	first phrase of a composition
*pakaḍ*	generally accepted musical phrase (or set of phrases) thought to encapsulate the essence of a particular *rāg*
*palṭā*	permutational routines of notes sung to *sargam* syllables (*sa re ga ma pa da ni*) or *ākār*
*riyāz*	musical practice (in contrast to *tālīm*—musical training)
*rūpak tāl*	7 beat *tāl*
*sam*	first beat of a rhythmic cycle
*Sāṅgīt Visharad*	lengthy text detailing all the information that a student should know to pass the qualification of the same name. School music teachers are usually required to have this qualification
*sargam*	syllables used to name the notes of a scale in a *rāg*
*saptak*	octave
*śruti*	microtone
*śruti box*	common name for an electronic *tanpūrā*
*śiṣya*	a pupil learning a craft from a *guru*
*sthāyī*	first section of a *bandiś*
*svar*	musical note
*tablā*	pair of drums used widely in modern North Indian Classical music styles
*tāl*	musical metre (the name given to rhythmic cycles)
*tālīm*	musical training, instruction or education (in contrast to *riyāz*—music practice)
*tān*	precomposed or improvised phrases interspersed with sections of the *sthāyī* and *antarā* phrases of the *bandiś* (see also *bol tān*) sung in *ākār* or using *sargam* syllables
*tānpūrā*	long necked plucked string instrument used for creating a drone
*ṭhāt*	heptatonic scale/mode
*ṭhumrī*	light romantic form of North Indian Classical music (in this context, ‘light’ does not denote ‘simplified’)
*tihāi*	polyrhythmic technique found in Indian classical music where a phrase is repeated three times, often used to conclude a piece
*tīn tāl*	16 beat cycle played on a percussion instrument
*tīvră*	sharpened note (only applied to ma)
*upaj*	extemporisation or variations on a theme
*vibhāg*	duration of rhythmic phrasing within a *tāl*
*vistār*	expansion or elaboration of a musical phrase or *rāg*
*yaman*	name of a *rāg* in *kalyāṇ ṭhāṭ*, often taught in the beginning stages of a student’s training
*yaman kalyān*	name of a *rāg* like *yaman*, except that in the descent, it gently touches the flat ma using a ga ma ga pattern
